# Supplementary figures and images for: PTRN-1 (CAMSAP) and NOCA-2 (NINEIN) are required for microtubule polarity in Caenorhabditis elegans dendrites
Source: PLoS Biol. 2022 Nov 17;20(11):e3001855. doi: 10.1371/journal.pbio.3001855 (PMC9714909; doi:10.1371/journal.pbio.3001855)

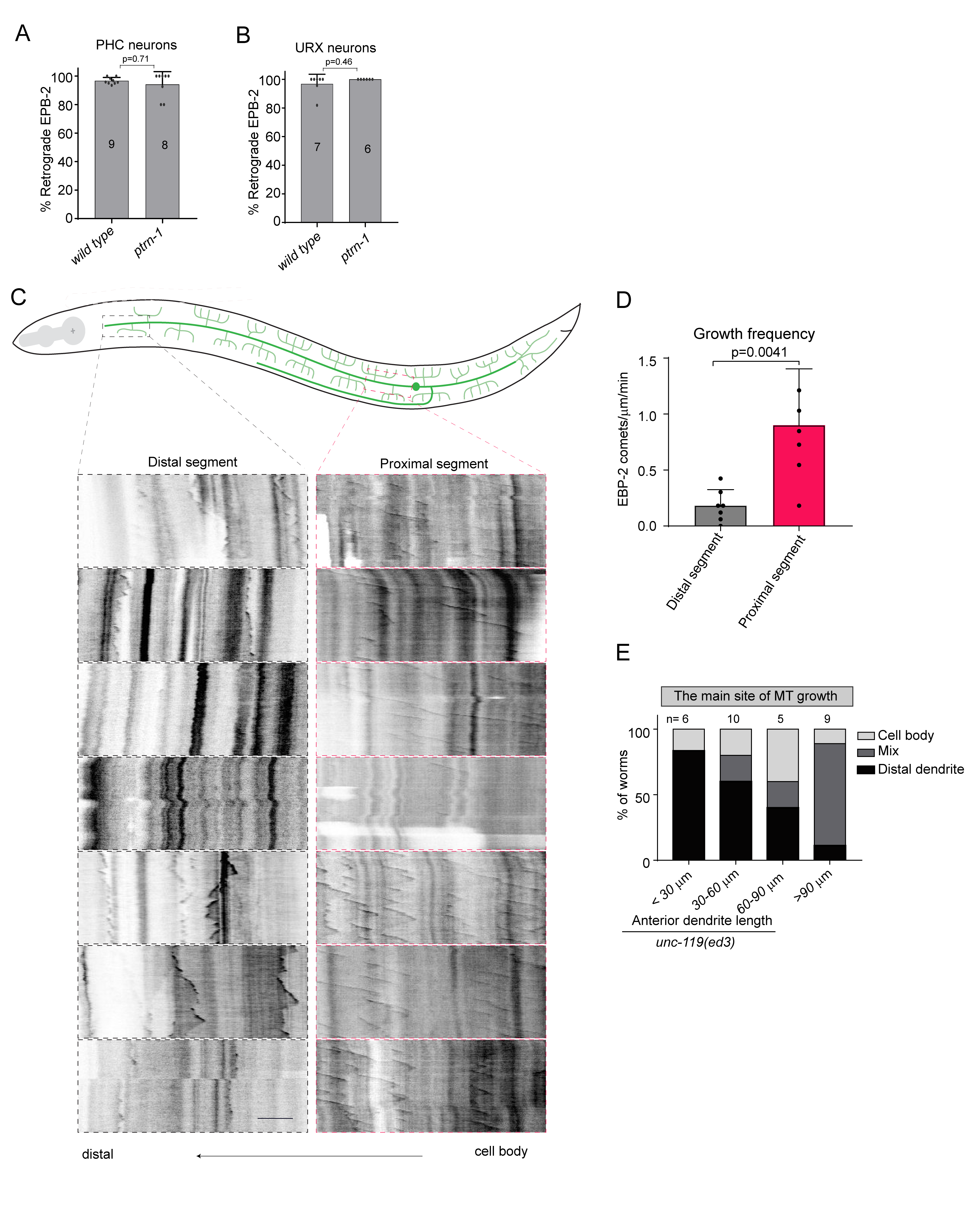

Supplement: S1 Fig — (A, B) Quantification of the percentage of retrograde EBP-2::GFP growth events in the ciliated PHC dendrites (A) and the nonciliated URX dendrites (B) in wild-type and the ptrn-1 mutant. Error bars represent SD; statistical analysis is followed by unpaired Student t test. Number of analyzed animals is indicated. (C, D) Examples of EBP-2::GFP dynamics in the mature PVD neuron imaged in the distal and proximal anterior dendrite (C) and the quantification of EBP-2 growth frequency (D). Error bars represent SD; statistical analysis was performed with an unpaired Student t test. (E) The quantification of the main site of microtubule growth during neuron developing in the unc-119 mutant visually classified as mainly in the distal dendrite (distal dendrite), throughout the dendrite (mix) or as coming from the cell body (cell body). The data underlying the graphs shown in the figure can be found in S1 Data. (TIF) [file pbio.3001855.s001.tif]

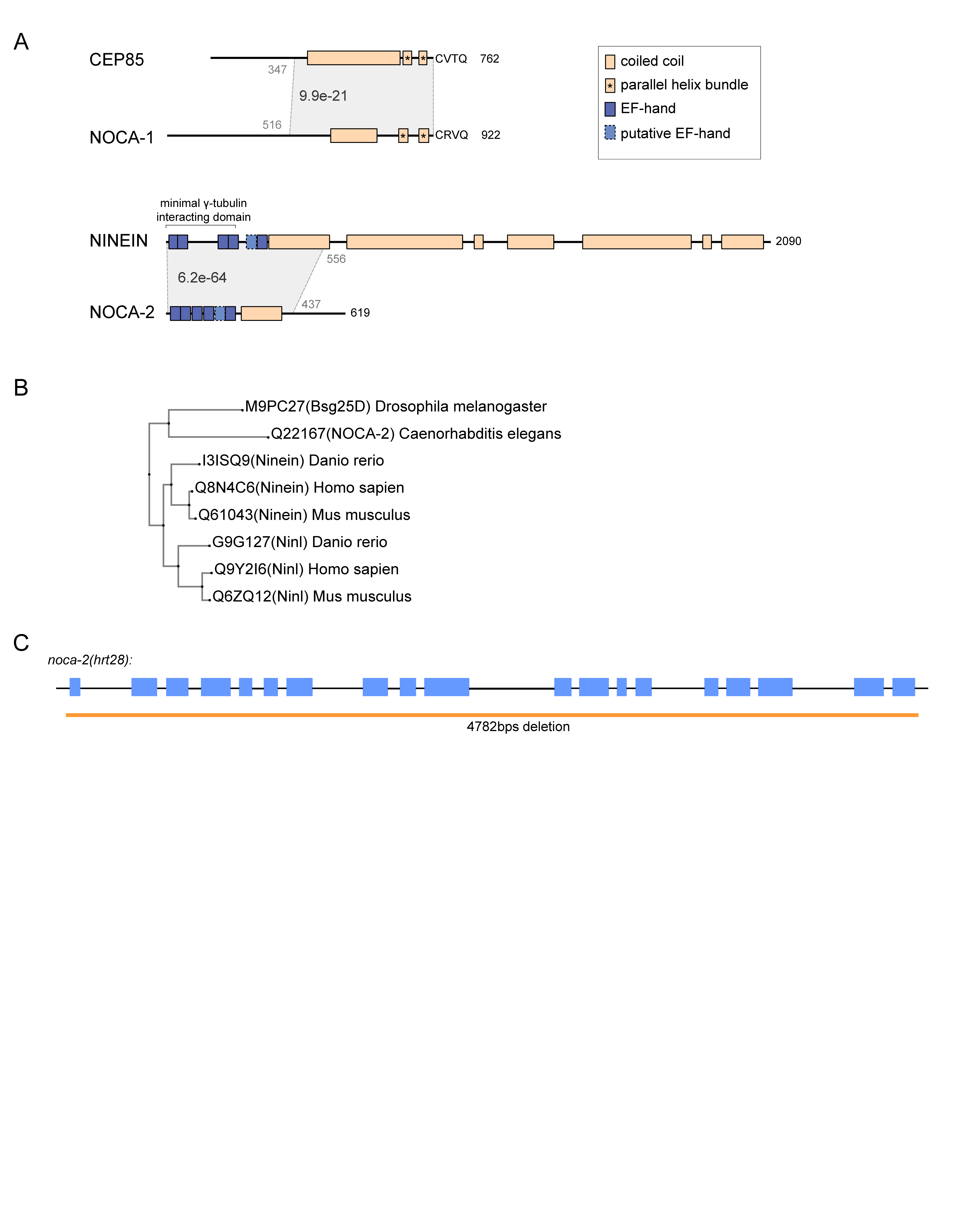

Supplement: S2 Fig — (A) Schematic representation of NOCA-1, NOCA-2, and human NINEIN and CEP85 based on UniProt annotations and Alphafold2 predicted 3D structure. NOCA-2 and NINEIN are unambiguous orthologs as evidenced by phylogenetics (B) and domain composition, but the evolutionary relation between NOCA-1, NOCA-2, and NINEIN is not so obvious despite initial reports that NOCA-1 and NINEIN are orthologs [48]. Alphafold2 predictions of NOCA-1, as available at https://alphafold.ebi.ac.uk/entry/G5EEK3, reveal no EF-hands or other globular domains that would normally allow unambiguous establishment of homology with NINEIN. Instead, they reveal a large coiled coil and a C-terminal set of parallel interacting alpha helices (marked with *). A profile search of this C-terminal region of NOCA-1 versus a database of profiles of human proteins hits CEP85 as best hit with e-value 0.0085 using HHPRED at the MPI-Toolkit on July 4. Reciprocal searches of full-length CEP85 profiles versus profiles of all C. elegans proteins hit NOCA-1 profile with 9.9e-21. These profile searches thus reveal that NOCA-1 and CEP85 are bidirectional best hits. In addition, Alphafold2 predicted structures of CEP85 reveal a similar set of interacting alpha helices in their C-term as predicted for NOCA-1, which is absent from NOCA-2 and NINEIN. Finally, both CEP85 and NOCA-1 share a C-terminal CxxQ putative farnesylation motif, which is also absent from NOCA-2 and NINEIN. Based on the reciprocal best profile-profile hits, structural and motif similarities, it is likely that CEP85 and NOCA-1 are homologs and that NOCA-1 is not homologous to NINEIN nor to NOCA-2. (B) Phylogenetic tree of full-length NINEIN and NINEIN Like (Ninl) proteins from various species. (C) Gene structure of the noca-2 gene and the hrt28 deletion. (TIF) [file pbio.3001855.s002.tif]

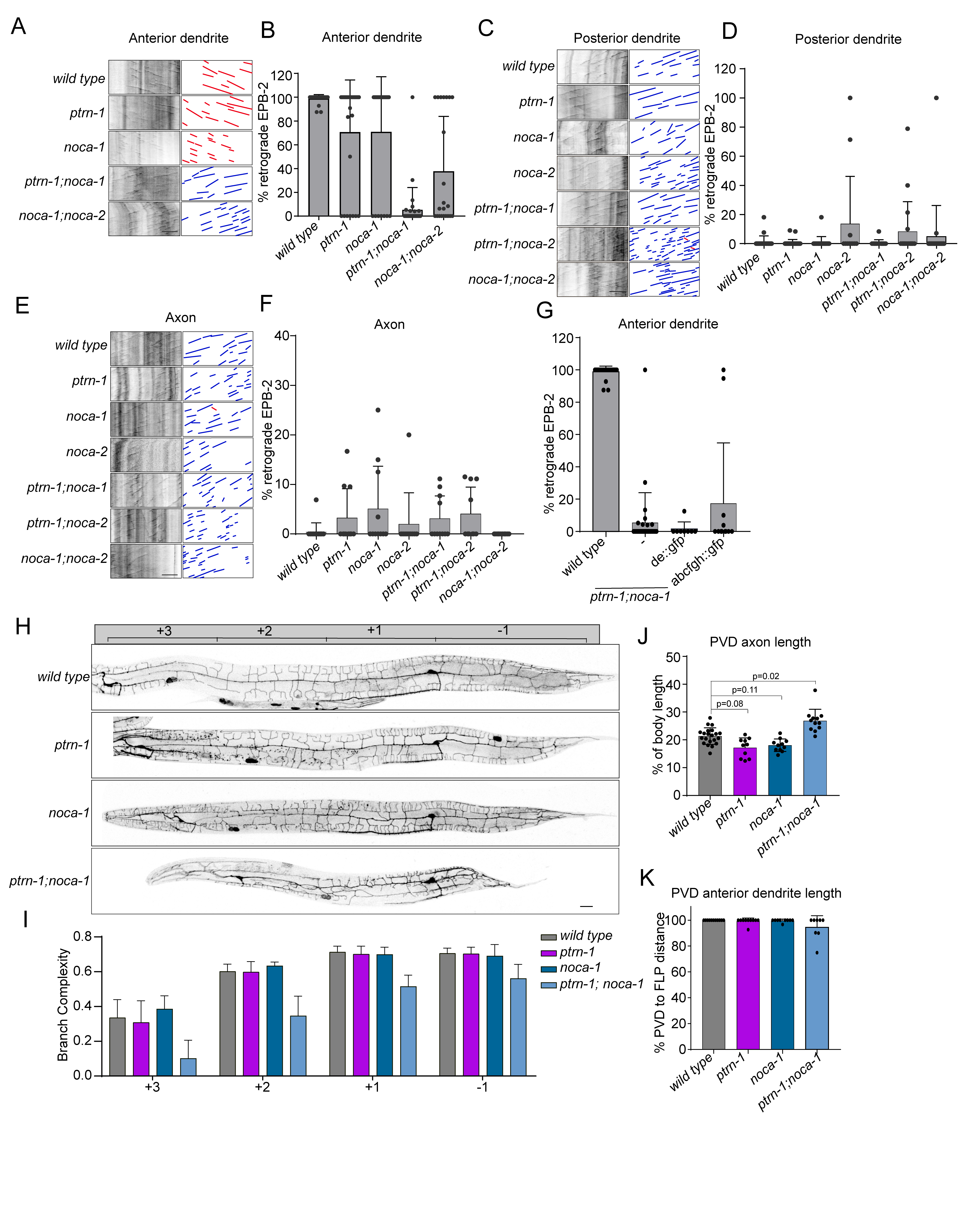

Supplement: S3 Fig — (A-F) Representative kymographs and quantification of microtubule polarity in the mature PVD neurons in the indicated mutants using EBP-2::GFP. The percentage of retrograde growing events in the anterior dendrite (A, B), in the posterior dendrite (C, D), and in the axon (E, F). Scale, 5 μm. (G) Quantification microtubule polarity using EBP-2::GFP in the anterior dendrite of wild-type and ptrn-1;noca-2 mutants with or without 2 tagged NOCA-1 rescue constructs [48]. (H-J) Quantification of the PVD morphology. (H) Representative examples of the PVD morphologies in the indicated mutants. Scale, 20 μm. Quantification of (I) the PVD dendritic branch complexity [45]; (J) the relative axon length in the ventral nerve cord; (K) the relative length of the anterior dendrite. For microtubule polarity analysis, the animals were from L4 to young adult stage. For PVD morphology analysis, only young adult stage animals were analyzed. Error bars represent SD; statistical analysis, Kruskal–Wallis test followed by Dunn’s multiple. The data underlying the graphs shown in the figure can be found in S1 Data. (TIF) [file pbio.3001855.s003.tif]

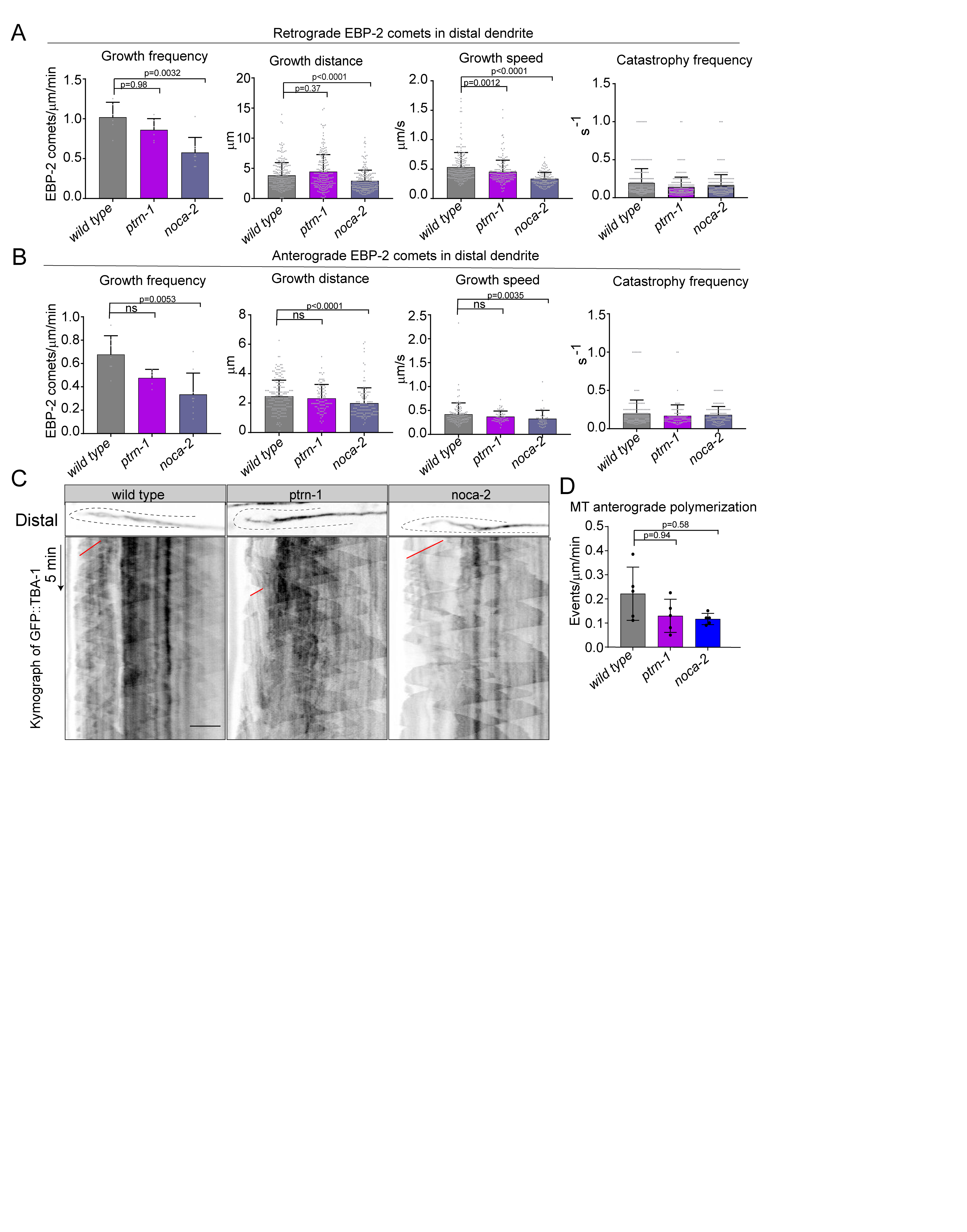

Supplement: S4 Fig — (A, B) Quantification of retrograde (A) and anterograde (B) microtubule plus-end dynamics in the distal 20 μm of the anterior PVD dendrite during neuron development using the plus tip marker EBP-2::GFP. For ptrn-1 and noca-2 mutants, only animals that retained distal microtubule nucleation were quantified. For speed measurements, only growth events of >2 μm were considered. Scale, 5 μm. Error bars represent SD; statistical analysis, Kruskal–Wallis test followed by Dunn’s multiple comparisons test. Number of analyzed animals is indicated. (C) Representative kymographs of GFP::TBA-1 in the distal anterior dendrite. Red lines, growing plus-end out MTs in distal region. Scale bar, 5 μm. The distal anterior PVD dendrites are indicated with dashed lines. (D) Quantification of plus-end out microtubule polymerization frequencies in the distal region of the growing anterior dendrite. Only animals that displayed distal microtubule nucleation were considered. Error bars represent SD; statistical analysis, Kruskal–Wallis test followed by Dunn’s multiple comparisons test. Five animals for each group were analyzed. The data underlying the graphs shown in the figure can be found in S1 Data. (TIF) [file pbio.3001855.s004.tif]

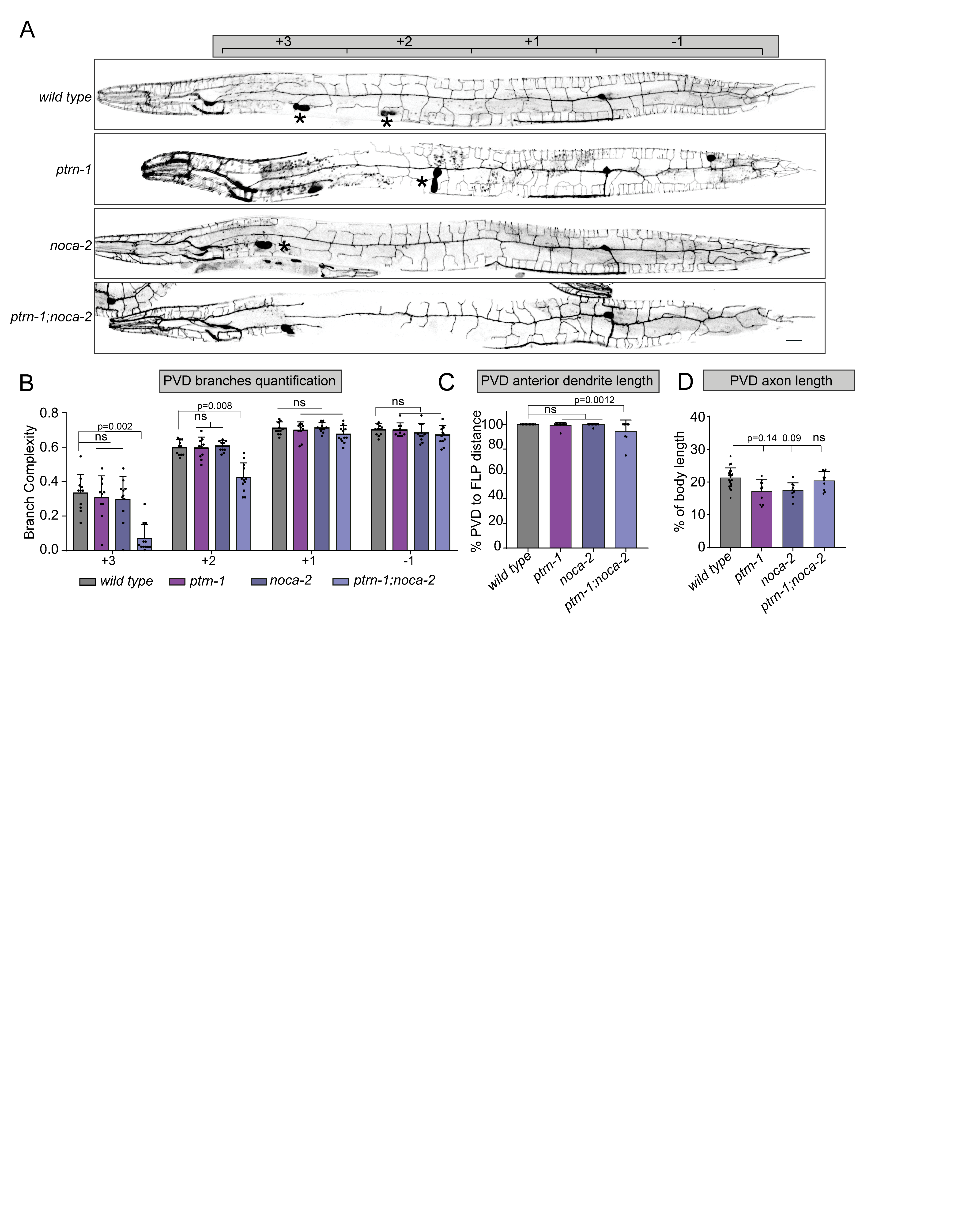

Supplement: S5 Fig — (A) Representative images of the PVD morphologies. Note that this marker is also expressed in another neuron (FLP) located in the head (left of the +3 region) and also in the coelomocytes (marked with *), which were used as injection marker. (B) Quantification of PVD dendritic branch complexity in the 4 PVD regions along the anteroposterior axis as indicated in (A), based on [45]. (C) Quantification of the anterior dendrite outgrowth towards the FLP cell body localized in the head. (D) Quantification of the relative axon length in the ventral nerve cord. Scale, 20 μm. Analyzed animals were young adult stage; Error bars represent SD; statistical analysis, Kruskal–Wallis test followed by Dunn’s multiple comparisons test. The data underlying the graphs shown in the figure can be found in S1 Data. (TIF) [file pbio.3001855.s005.tif]

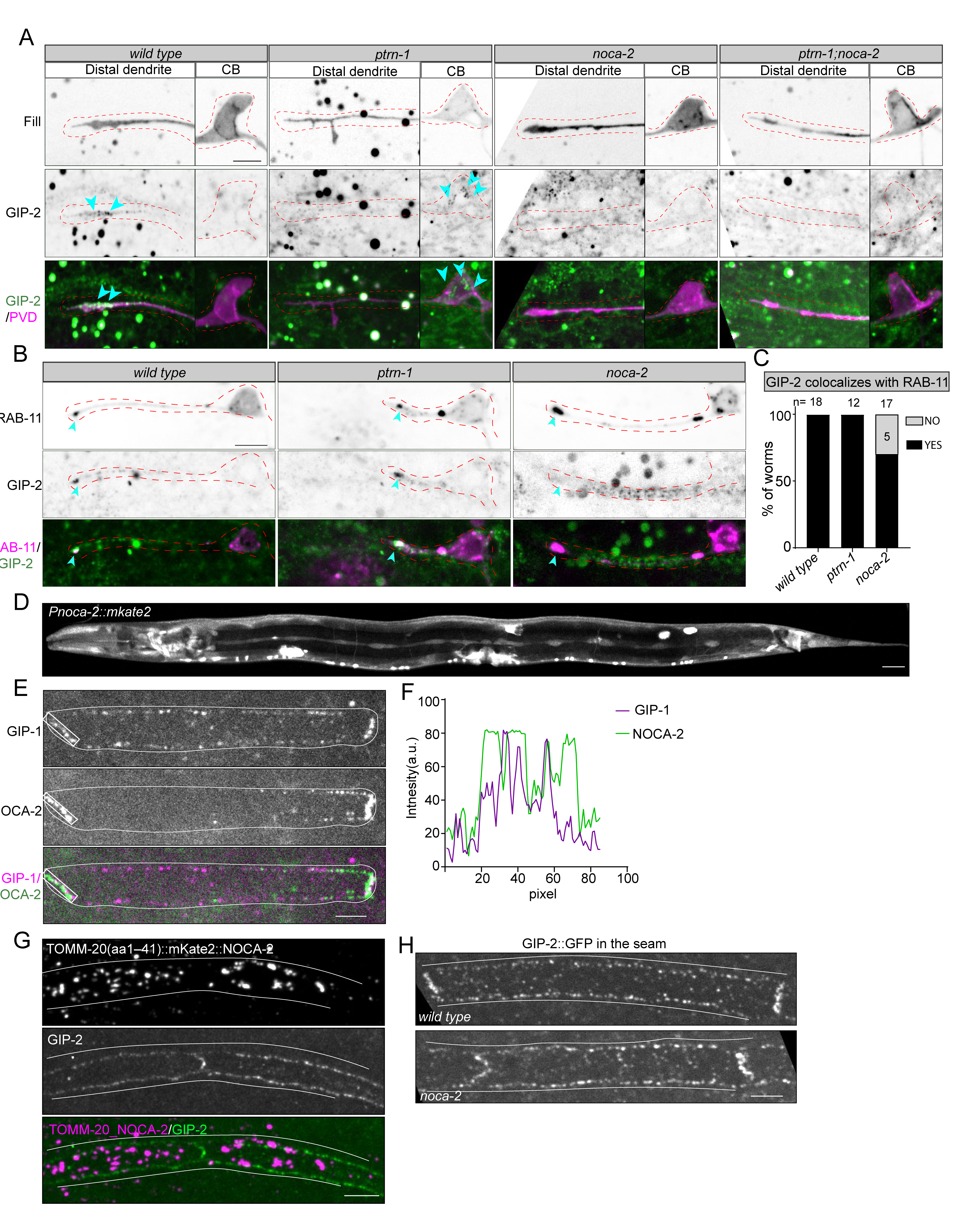

Supplement: S6 Fig — (A) Representative examples of endogenously tagged GIP-2::GFP in the PVD neuron. GIP-2 accumulated in the cell body in the ptrn-1 mutant (second panel) and without obvious GIP-2 accumulation in noca-2 mutant (third panels) and ptrn-1;noca-2 mutants (last panels). Green: GIP-2, magenta: PVD neuron fill. GIP-2 puncta are indicated with blue arrowheads. Scale, 5 μm. The developing neurons are indicated with red dashed lines. (B) Example images of mKate2::RAB-11 (magenta) and GIP-2::GFP (green) colocalization in the distal segment of the growing PVD anterior dendrite. The localization of RAB-11 and GIP-2 in developing anterior dendrite is indicated with arrowheads. Scale, 5 μm. The PVD neuron is indicated with red dashed lines. (C) Quantification of the number of animals in which mKate2::RAB-11 colocalizes with GIP-2::GFP in the distal segment of the growing PVD anterior dendrite; gray: the percentage of animals that have RAB-11 accumulated in distal dendrites but without GIP-2 accumulation. Number of analyzed animals is indicated. (D) The expression pattern of mKate2 driven by 2 kb of the noca-2 promoter sequence. Scale, 20 μm. (E, F) Examples of localization (E) and the intensity quantification (F) of endogenous NOCA-2 and GIP-1 in epidermal seam cells. Scale, 5 μm. The epidermal seam cells are marked with white lines. (G) Representative example images of the GIP-2 (green) localization upon artificial NOCA-2 (magenta) mislocalization to mitochondria by fusing it to TOMM-20 (1–41 amino acids). Scale, 5 μm. The epidermal seam cells are marked with white lines. (H) The localization of GIP-2 in epidermal seam cells in wild-type (upper panels) and in noca-2 mutant (lower panels). Scale, 5 μm. The epidermal seam cells are marked with white lines. The data underlying the graphs shown in the figure can be found in S1 Data. (TIF) [file pbio.3001855.s006.tif]

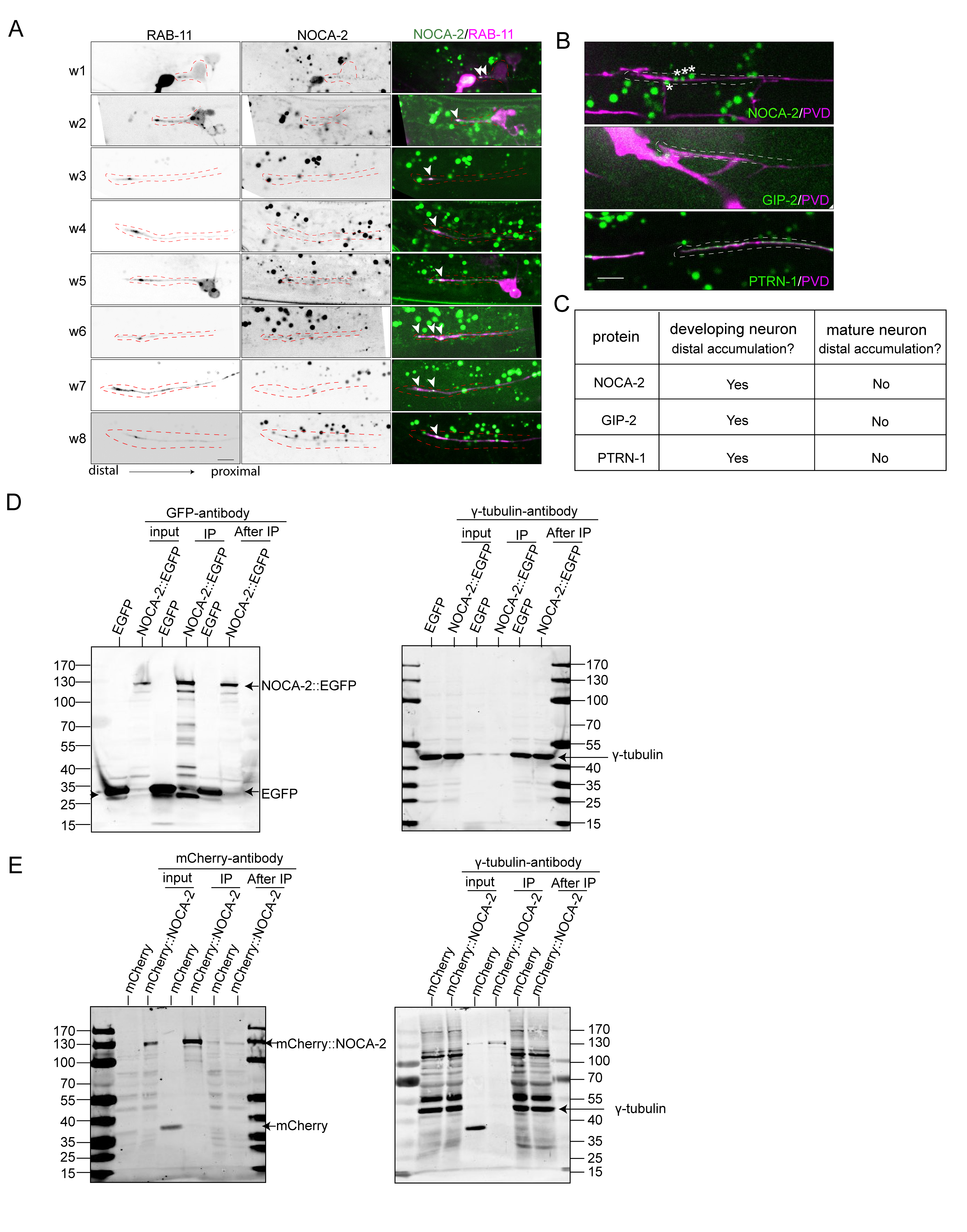

Supplement: S7 Fig — (A) Examples of endogenous NOCA-2::GFP (green) and PVD expressed mKate2::RAB-11 (magenta) at different developmental stages of PVD anterior dendrite. The colocalization is indicated with arrowheads in the merged image and the outline of the dendrite is marked by a red dashed line. Scale, 5 μm. (B, C) Representative example of endogenously tagged NOCA-2 and GIP-2 and PVD expressed PTRN-1 in the distal part of the mature PVD anterior dendrite (B) and a summary diagram of localization of localization of NOCA-2, GIP-2, and PTRN-1 in distal mature dendrites and developing dendrites (C). The distal anterior PVD dendrites are indicated by a dashed red line; * marks autofluorescent gut granules. Scale, 5 μm. (D) Pull-down of NOCA-2::EGFP and EGFP (control) from HEK293T cells using GFP-Trap magnetic beads. Anti-GFP and anti-γ-tubulin were used to detected NOCA-2 and GFP (left panel) and human γ-tubulin (right panel). (E) Streptavidin pull-down assays from HEK293T cells coexpressing with bio-mCherry-NOCA-2 or bio-mCherry with BirA. Anti-mCherry and anti-γ-tubulin were used to detected NOCA-2 or mCherry (left panel) and human γ-tubulin (right panel). (TIF) [file pbio.3001855.s007.tif]

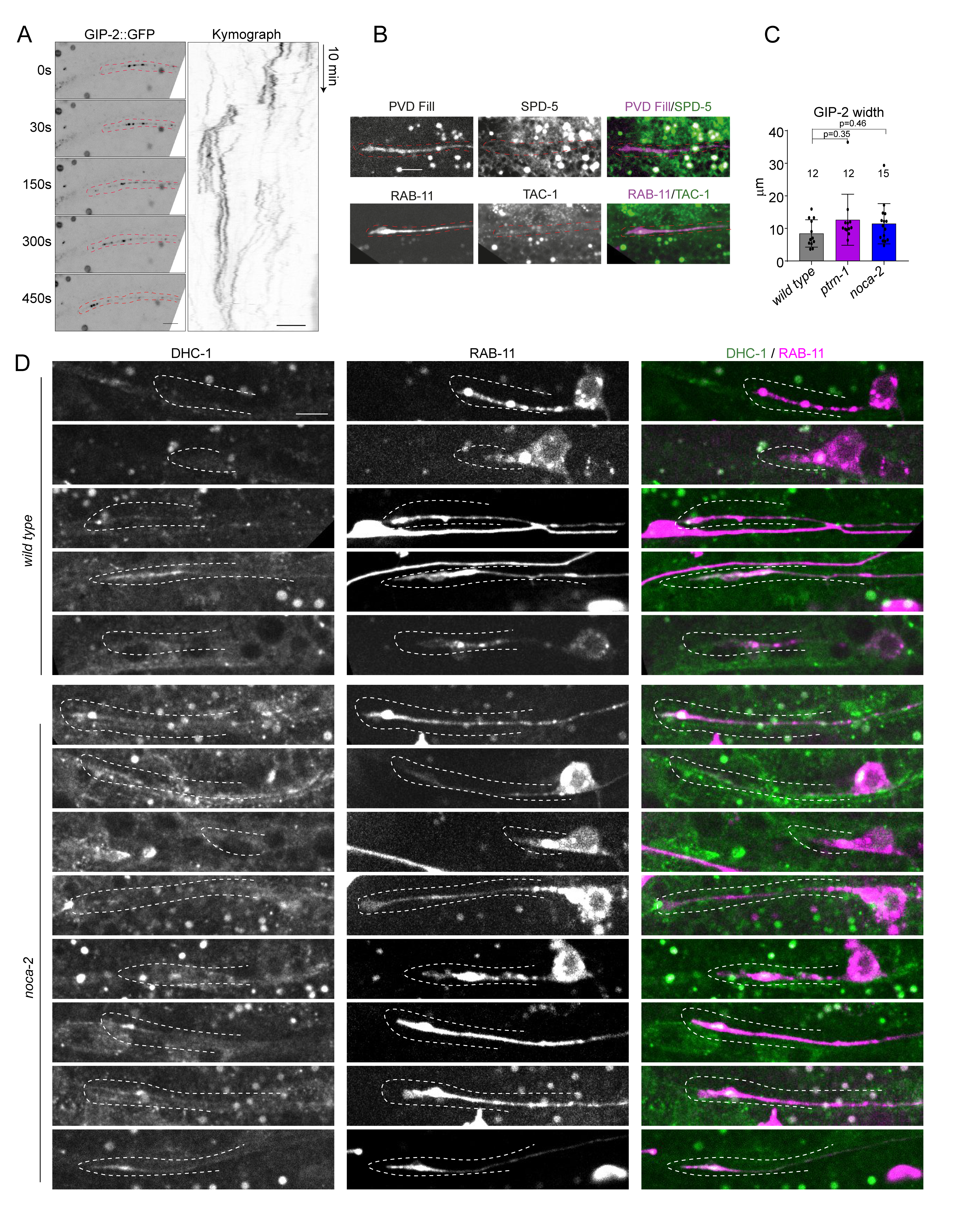

Supplement: S8 Fig — (A) Stills and kymograph of endogenous GIP-2::GFP localization over time (left panel) in growing PVD anterior dendrite. Scale, 5 μm. The distal dendrite is indicated with red dashed lines. (B) GFP::SPD-5 and GFP::TAC-1 localization at the growing PVD anterior dendrite using endogenously tagged strains. (C) Quantification of GIP-2 cluster width in the growing PVD anterior dendrite. (D) Multiple examples of endogenous DHC-1::GFP (green) and PVD expressed mKate2::RAB-11 (magenta) in the growing PVD anterior dendrite. Scale, 5 μm. The distal anterior PVD dendrites are indicated with white dashed lines. The data underlying the graphs shown in the figure can be found in S1 Data. (TIF) [file pbio.3001855.s008.tif]

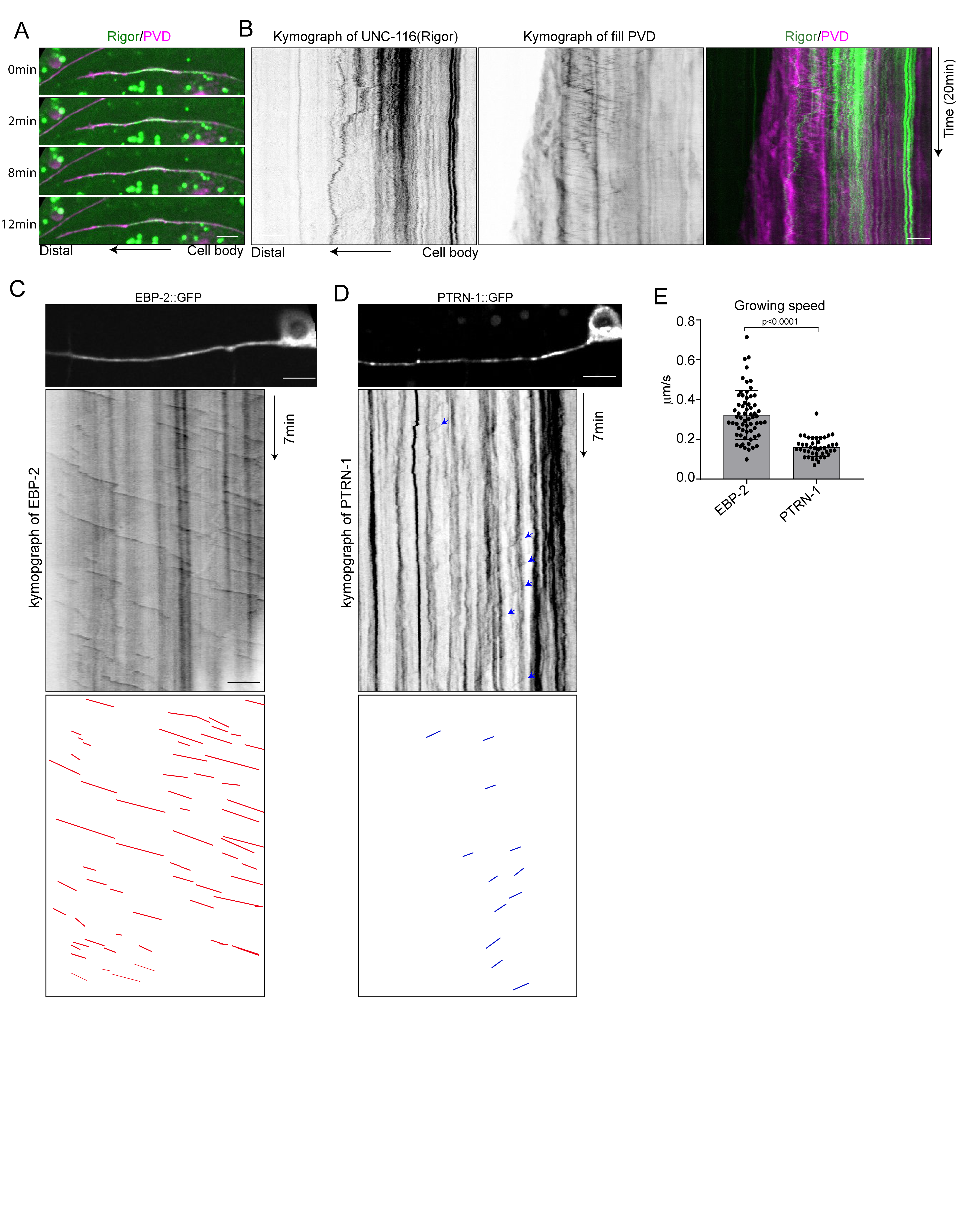

Supplement: S9 Fig — (A, B) Stills (A) and kymographs (B) of UNC-116(rigor)::GFP localization in the PVD anterior dendrites (A, green). Myristoylated mKate2 was used as a fill (magenta). Scale, 5 μm. (C, D) Representative example kymograph of EBP-2::GFP (C) and mKate2::PTRN-1 (D) in the mature PVD anterior dendrite. Examples of moving PTRN-1 puncta are indicated with blue arrowheads. Scale, 5 μm. (E) Quantification of EBP-2::GFP growth speed and PTRN-1 moving speed in the mature PVD anterior dendrite. Error bars represent SD; statistical analysis is followed by unpaired Student t test. The data underlying the graphs shown in the figure can be found in S1 Data. (TIF) [file pbio.3001855.s009.tif]

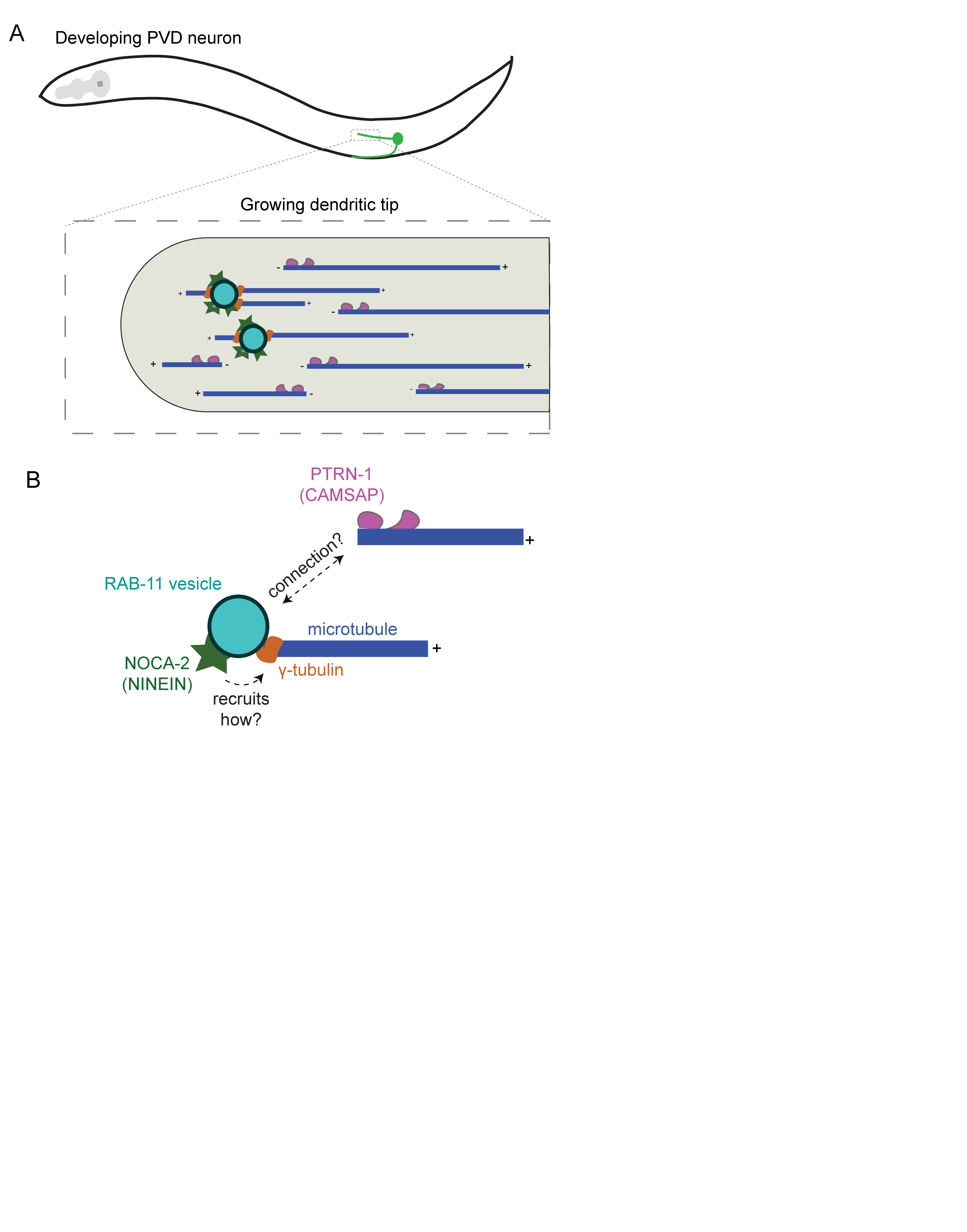

Supplement: S10 Fig — (A, B) Model for NOCA-2 (NINEIN) and PTRN-1 (CAMSAP) functioning at the growing dendrite tip of the PVD neuron. NOCA-2 localizes to the MTOC endosomes localized to the dendrite tip and is involved in recruiting the microtubule nucleating γ-tubulin, whereas PTRN-1 localizes around the MTOC vesicles where it may stabilize the nucleated microtubules. (TIF) [file pbio.3001855.s010.tif]
