# Supplementary material for: PTRN-1 (CAMSAP) and NOCA-2 (NINEIN) are required for microtubule polarity in Caenorhabditis elegans dendrites
Source: PLoS Biol. 2022 Nov 17;20(11):e3001855. doi: 10.1371/journal.pbio.3001855 (PMC9714909; doi:10.1371/journal.pbio.3001855)

Original Blots

RAW BLOT Figure S7D (left) **RAW BLOT Figure S7E (right)**

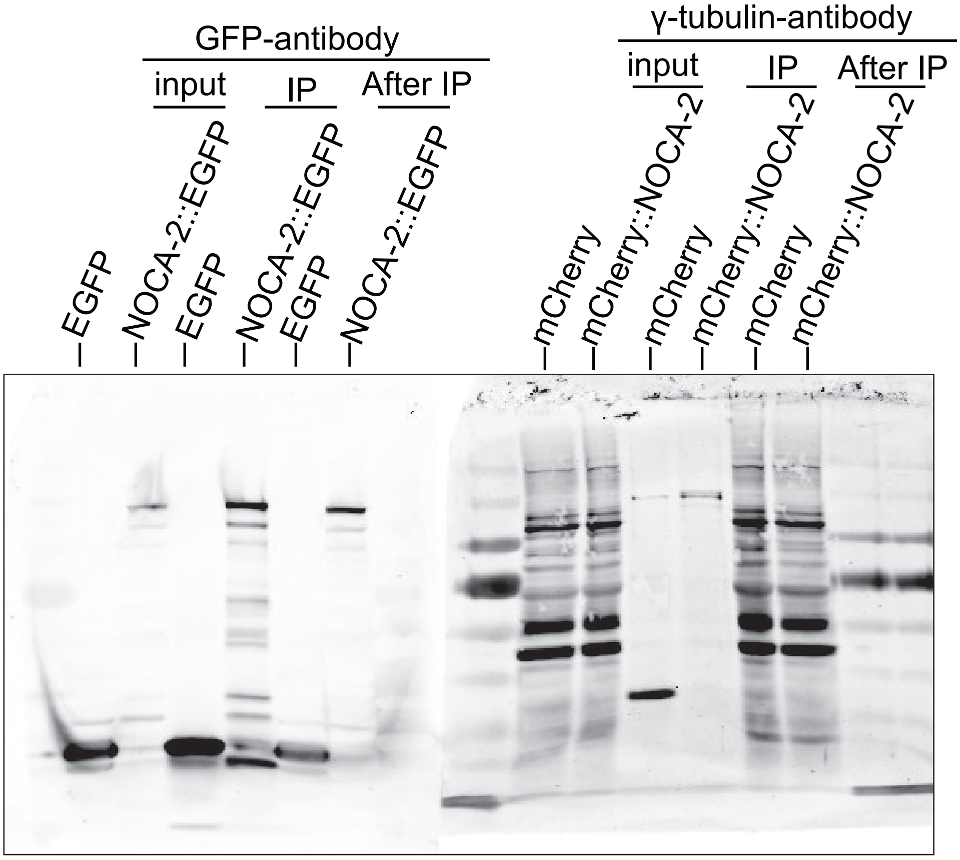

RAW BLOT Figure S7E (left)
(image is flipped)

RAW BLOT Figure S7D (right)

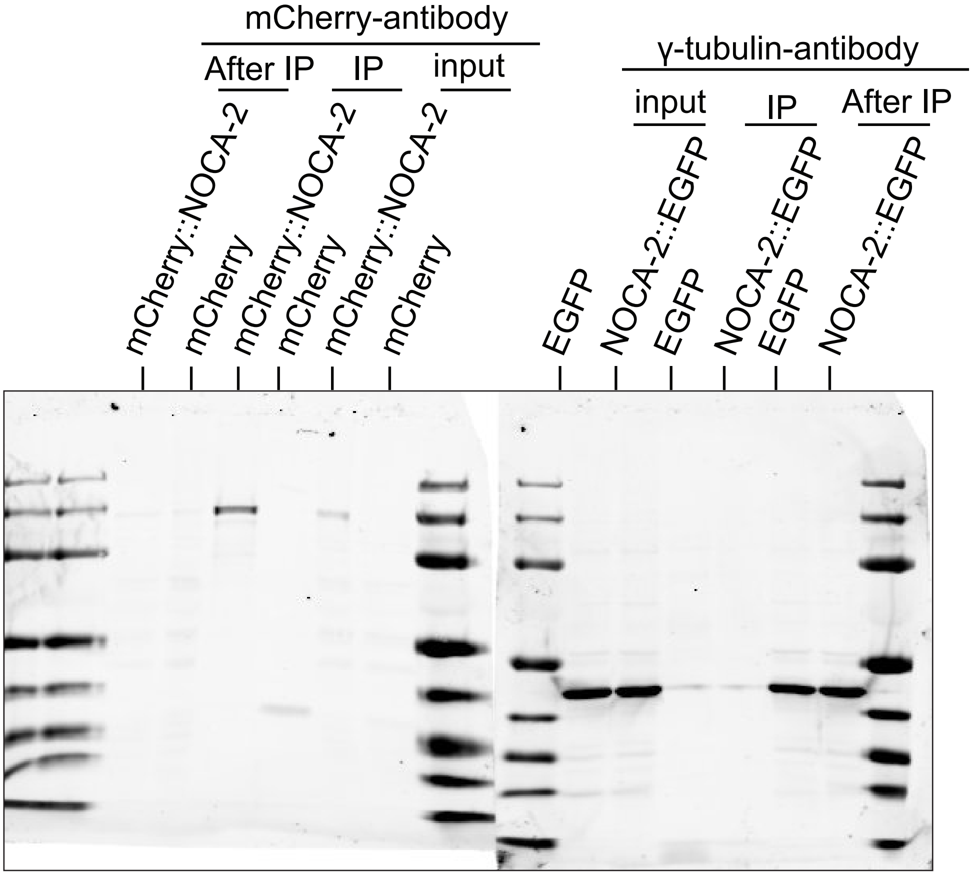

Supplement: S1 Raw Images — (PDF) [file pbio.3001855.s018.pdf]
