# Supplementary material for: PTRN-1 (CAMSAP) and NOCA-2 (NINEIN) are required for microtubule polarity in Caenorhabditis elegans dendrites
Source: PLoS Biol. 2022 Nov 17;20(11):e3001855. doi: 10.1371/journal.pbio.3001855 (PMC9714909; doi:10.1371/journal.pbio.3001855)
Supplement: Attachment — Submitted filename: 122546_1_rebuttal_2281735_r6mbbj.docx [file pbio.3001855.s019.docx]

**Manuscript number:** #RC-2021-01191

**Corresponding author(s):** Martin Harterink

## 1. General Statements [optional]

## 2. Description of the planned revisions

Reviewer #1

Most conclusions are supported by the data, except for when the authors say: "Taken together, these results show that PTRN-1 (CAMSAP) and NOCA-2 (NINEIN) act in parallel in the PVD neuron during early development to establish minus-end out microtubule organization, and that this organization is important for proper dendritic morphogenesis." But the authors show that removal of Patr results in some neurons having a complete anterograde phenotype in the anterior genotype, but that no Patr neurons have a severe morphology defect (Fig 2). This would suggest that the severe morphology defects in Patr/Noca-2 double mutants are not simply due to the reversal of polarity in the anterior dendrite. This should be discussed.

*We agree with the comment, and we will discuss this more clearly in a revised manuscript.*

The paper could be strengthened with some biochemistry showing that Noca-2 can associate with γ-TuRCs i.e. do purified fragments of Noca-2 pull out γ-TuRCs from a cell extract (not necessarily a neuron cell extract)? This should be possible within 1 month.

*We thank the reviewer for this suggestion. We will perform some biochemistry experiment to probe the association of NOCA-2 with γ-TuRCs. However, instead of doing the IP by overexpression of NOCA-2 and γ-TuRCs in cells, we will use the CRISPR knockin animals for NOCA-2 and γ-TuRCs, to exclude potential overexpression artifacts.*

*Minor comments*

*1) "However, in polarized cells such as neurons, most microtubules are organized in a non-centrosomal manner (Nguyen et al., 2011)." Need more up to date reference here, such as a recent review from Jens Lüders.*

*We will update the references in the revision version of the manuscript.*

*2) "and also in Drosophila Patronin was found important for dendritic microtubule polarity (Feng et al., 2019)." Also Wang et al., 2019 in eLife.*

*We will add this reference.*

*3) "In the non-ciliated PHC neuron or the ciliated URX neuron we did not observe microtubule organization defects in the ptrn-1 mutant (Supplemental figure 1A-B), which suggests that these neurons do less or do not dependent on PTRN-1." End of sentence needs re-phasing*

*We will rephrase the text.*

Reviewer #2

1. NOCA-2 is described as a previously unidentified member of the ninein family. In order to evaluate this claim critically, it would be helpful to have a figure showing how similar NOCA-1 and NOCA-2 are to mammalian ninein. It is also critical to include a phylogeny to get a better feel for how NOCA-2 fits into the evolutionary history of the family.

*We agree with the suggestion. We will perform this analysis and add it to a revised version of the manuscript.*

2. The nucleation assay used throughout is not very clear as one reads through the manuscript. The color-coding of Fig 1G could be better defined in the legend, and it would be helpful to have more information in the legend or results about what is meant here by microtubule nucleation. Is it simply initiation of new microtubule growth events? If so, how are these distinguished from catastrophe rescue? It would be good to use the same color coding in 2E.

*We thank the reviewer for pointing this out. In Fig 1G and 2E we indeed quantified EBP-2::GFP growth events. Although we later show that the microtubule nucleator gamma-tubulin localized to the distal segment where we observe increased microtubule growth events, we agree that we cannot distinguish microtubule nucleation from regrowth after catastrophe. Therefore, we will describe this more accurately in the text, legend and in the figure.*

3. The colocalization of Rab-11 and NOCA-2 seems to be supported only by a single overlapping puncta in a neuron before the anterior dendrite extends (Fig 4). It would be good to flesh out this data set more as it is an important part of the argument that NOCA-2 is involved in recruiting g-TuRC to Rab11 endosomes.

*We thank the reviewer for pointing this out. We will flesh out this data either by adding several examples and/or a movie to show the localization of Rab-11 and NOCA-2 in the revised version of the manuscript.*

4. Summary diagrams of results either as conclusions are made in the individual figures or synthesized at the end would help readers to understand the evidence that NOCA-2 and PTRN-1 function at different steps in establishment of MT polarity

*We agree that a summary diagram could be helpful, and we will consider adding this to the revised version of the manuscript.*

6. It would be helpful to clearly state at some point which aspects of localization that are described are seen only in developing dendrites and which are seen in both developing and mature dendrites. For example, is there any similarity in localization of PTRN-1 NOCA-2 and gip2 in mature dendrites to that shown in immature? Is there any sign of continued localization to RAB11 vesicles, or is this only transient? Perhaps a diagram to summarize these findings would also be helpful.

*We thank the reviewer for pointing this out. We will better explain the localization of NOCA-2, PTRN-1, GIP-2 and RAB-11 vesicles in developing neurons vs mature neurons in a revised version of the manuscript.*

7. The authors propose several different ideas about how Patronin might contribute to Rab11 vesicle localization. However, I am not sure that they really describe the simplest one: that Patronin helps minus ends grow out from the cell body as shown in Drosophila (and Fig S6 here), and that these minus-end-out microtubules could be the tracks used to transport Rab11 into dendrites. Have I missed some reason why this model is not presented as a good fit for the data?

*We thank the reviewer for pointing this out. Feng et al indeed showed that EB proteins can track microtubule plus- and minus-end growth in the sensory neurons of Drosophila. Since the slower event co-localize with Patronin they suggested that these help to populate the minus-end out microtubules in the drosophila dendrites (Feng et al., 2019). Although we do not have strong data against this model for the PVD dendrites in C. elegans, there are several observations that to us suggest that it is unlikely that minus-end growth is the driving force for the forward movement of the MTOC vesicles. These include: the MT being mixed in the distal segment, therefore it is hard to imagine how specifically one pool is growing; we do not see EBP-2 localize to the Camsap puncta as was seem in Drosophila; the Camsap dynamics at the growth cone seem very different (less processive) to the dynamics in the shaft (which indeed could be minus-end growth). We will make this reasoning clearer in the revised manuscript.*

8. There are some grammatical errors throughout, as well as a few typos (like PTNR-1 for PTRN-1).

*We will correct the text grammar and typos in the revision version of manuscript.*

## 3. Description of the revisions that have already been incorporated in the transferred manuscript

## 4. Description of analyses that authors prefer not to carry out

Reviewer 2:

5. NOCA-1 is introduced at the beginning of the manuscript and appears to act in parallel to both NOCA-2 and PTRN-1. One is left with many questions, for example, is it also required to recruit g-TuRCs to Rab11 vesicles, or does it have some other role? However, I appreciate that it is beyond the scope of a single manuscript to answer all questions and the authors state a clear rationale for focusing on NOCA-2.

*We agree that the function of NOCA-1 is interesting to be investigated in the future, since we found it acts redundantly to PTRN-1 and NOCA-2. As NOCA-1 is an essential gene this brings along some technical difficulties to properly address its function and would require generating novel tools. We appreciate the reviewers understanding that this is beyond the scope of the current manuscript.*
