# Supplementary material for: PTRN-1 (CAMSAP) and NOCA-2 (NINEIN) are required for microtubule polarity in Caenorhabditis elegans dendrites
Source: PLoS Biol. 2022 Nov 17;20(11):e3001855. doi: 10.1371/journal.pbio.3001855 (PMC9714909; doi:10.1371/journal.pbio.3001855)
Supplement: Attachment — Submitted filename: He et al. 2022 - Revisions.docx [file pbio.3001855.s021.docx]

Reviewer #1

Most conclusions are supported by the data, except for when the authors say: "Taken together, these results show that PTRN-1 (CAMSAP) and NOCA-2 (NINEIN) act in parallel in the PVD neuron during early development to establish minus-end out microtubule organization, and that this organization is important for proper dendritic morphogenesis." But the authors show that removal of Patr results in some neurons having a complete anterograde phenotype in the anterior genotype, but that no Patr neurons have a severe morphology defect (Fig 2). This would suggest that the severe morphology defects in Patr/Noca-2 double mutants are not simply due to the reversal of polarity in the anterior dendrite. This should be discussed.

*We agree with the observation of the reviewer that MT reversal seems is insufficient to lead to morphological defects. We now describe this in the text.*

The paper could be strengthened with some biochemistry showing that Noca-2 can associate with γ-TuRCs i.e. do purified fragments of Noca-2 pull out γ-TuRCs from a cell extract (not necessarily a neuron cell extract)? This should be possible within 1 month.

*We thank the reviewer for this suggestion. We first tried to perform immunoprecipitation experiments from worms using endogenously tagged proteins. However due to the very low expression of these proteins we were not able to successfully perform this experiment. Therefore we have performed the experiment as suggested to probe the association of NOCA-2 with γ-TuRCs. However, we did not detect an interaction. We have added the results of the pull down experiments in supplemental figure 6D-E and discuss this result in our revision manuscript.*

*Minor comments*

*1) "However, in polarized cells such as neurons, most microtubules are organized in a non-centrosomal manner (Nguyen et al., 2011)." Need more up to date reference here, such as a recent review from Jens Lüders.*

*We have changed this reference for more up-to-date references in the revision manuscript.*

*2) "and also in Drosophila Patronin was found important for dendritic microtubule polarity (Feng et al., 2019)." Also Wang et al., 2019 in eLife.*

*We thank the reviewer for pointing this out. We have added this reference in the revision manuscript.*

*3) "In the non-ciliated PHC neuron or the ciliated URX neuron we did not observe microtubule organization defects in the ptrn-1 mutant (Supplemental figure 1A-B), which suggests that these neurons do less or do not dependent on PTRN-1." End of sentence needs re-phasing*

*We rephrased this text to:* “*In the non-ciliated PHC neuron and the ciliated URX neuron we did not observe microtubule organization defects in the ptrn-1 mutant (Supplemental figure 1A-B), indicating that the PTRN-1 requirement to organize the microtubules varies between neurons.”*

Reviewer #2

1. NOCA-2 is described as a previously unidentified member of the ninein family. In order to evaluate this claim critically, it would be helpful to have a figure showing how similar NOCA-1 and NOCA-2 are to mammalian ninein. It is also critical to include a phylogeny to get a better feel for how NOCA-2 fits into the evolutionary history of the family.

*We agree with the suggestion. We used online Sequence Manipulation Suite (*[*http://www.bioinformatics.org/sms2/ident_sim.html*](http://www.bioinformatics.org/sms2/ident_sim.html)*) to calculate protein similarity and identity of NOCA-1/2 to human NINEIN. We have added this analysis to the supplemental figure2C. Furthermore, to better understand how NOCA-2 fits into the evolutionary history of the family, we included a phylogenetic tree of the full length proteins by using MAFFT alignment (http://mafft.cbrc.jp/alignment/server/) (Katoh and Standley, 2013) in supplemental figure 2B. This alignment shows that NOCA-2 is evolutionary closer to NINEIN.*

2. The nucleation assay used throughout is not very clear as one reads through the manuscript. The color-coding of Fig 1G could be better defined in the legend, and it would be helpful to have more information in the legend or results about what is meant here by microtubule nucleation. Is it simply initiation of new microtubule growth events? If so, how are these distinguished from catastrophe rescue? It would be good to use the same color coding in 2E.

*We thank the reviewer for pointing this out. In Fig 1G and 2E we indeed quantified EBP-2::GFP growth events. Although we later show that the microtubule nucleator gamma-tubulin localized to the distal segment where we observe increased microtubule growth events, we agree that we cannot distinguish microtubule nucleation from regrowth after catastrophe. Therefore, we have described this more accurately in the text, legend and in the figure. In addition, to avoid the confusion, we have changed the title of the figure to” the main site of microtubule growth” in Fig 1G and 2E and supplemental figure 2A. Moreover, the color coding of Figure 1G and supplemental figure 2A have been change to the same color coding as Figure 2E.*

3. The colocalization of Rab-11 and NOCA-2 seems to be supported only by a single overlapping puncta in a neuron before the anterior dendrite extends (Fig 4). It would be good to flesh out this data set more as it is an important part of the argument that NOCA-2 is involved in recruiting g-TuRC to Rab11 endosomes.

*We agree this is an important point and have now added 8 more examples of the colocalizations of NOCA-2 and RAB-11 at different stages of dendrite development in supplemental figure 6A. Moreover, we added a movie of colocalization in video 5.*

4. Summary diagrams of results either as conclusions are made in the individual figures or synthesized at the end would help readers to understand the evidence that NOCA-2 and PTRN-1 function at different steps in establishment of MT polarity

*We agree and added a model summarizing our findings how NOCA-2 and PTRN-1 functions in establishing minus-end out microtubule polarity (Supplemental Figure 9).*

5. NOCA-1 is introduced at the beginning of the manuscript and appears to act in parallel to both NOCA-2 and PTRN-1. One is left with many questions, for example, is it also required to recruit g-TuRCs to Rab11 vesicles, or does it have some other role? However, I appreciate that it is beyond the scope of a single manuscript to answer all questions and the authors state a clear rationale for focusing on NOCA-2.

*We agree that the function of NOCA-1 is interesting to be investigated in the future, since we found it acts redundantly to PTRN-1 and NOCA-2. As NOCA-1 is an essential gene this brings along some technical difficulties to properly address its function and would require generating novel tools. We appreciate the reviewers understanding that this is beyond the scope of the current manuscript.*

6. It would be helpful to clearly state at some point which aspects of localization that are described are seen only in developing dendrites and which are seen in both developing and mature dendrites. For example, is there any similarity in localization of PTRN-1 NOCA-2 and gip2 in mature dendrites to that shown in immature? Is there any sign of continued localization to RAB11 vesicles, or is this only transient? Perhaps a diagram to summarize these findings would also be helpful.

*We thank the reviewer for pointing this out. Previous study from Liang et al have showed GIP-2 was visible as soon as the anterior dendrite emerged but absent near the tip of mature dendrites (Liang et al.;2020.; eLife). To clearer describe the localization of NOCA-2, PTRN-1 and GIP-2 in developing neurons vs mature neurons, we have added some representative examples and a summary diagram in supplemental figure 6B-C. As the anterior dendrite tip overlaps with head neurons, it is hard to completely rule our NOCA-2 and PTRN-1 presence in the distal segment, however we do not see enrichment of these proteins as seen in a developing neuron. To validate the reduction in distal microtubule nucleation, we imaged EBP-2 dynamics in animals in the distal dendrites in mature neurons (supplemental figure 1C). This result showed that the EBP-2 growth frequency was dramatically decreased in the distal dendrites (supplemental figure 1D). This suggests that MTOC vesicles localize to the dendrite tip during development to organize dendritic microtubules minus-end out, but that this is not maintained in mature neurons.*

7. The authors propose several different ideas about how Patronin might contribute to Rab11 vesicle localization. However, I am not sure that they really describe the simplest one: that Patronin helps minus ends grow out from the cell body as shown in Drosophila (and Fig S6 here), and that these minus-end-out microtubules could be the tracks used to transport Rab11 into dendrites. Have I missed some reason why this model is not presented as a good fit for the data?

*We agree that minus-end outgrowth may take place in the dendrite shaft and could contribute to the microtubule organization and thus MTOC transport. However in the distal dendrite the PTRN-1 dynamics look very different, therefore we do not believe that these dynamics represent minus-end growth. We now described this more clearly.*

8. There are some grammatical errors throughout, as well as a few typos (like PTNR-1 for PTRN-1).

*We asked a native speaker to correct the grammar*
