# Supplementary material for: PTRN-1 (CAMSAP) and NOCA-2 (NINEIN) are required for microtubule polarity in Caenorhabditis elegans dendrites
Source: PLoS Biol. 2022 Nov 17;20(11):e3001855. doi: 10.1371/journal.pbio.3001855 (PMC9714909; doi:10.1371/journal.pbio.3001855)
Supplement: Attachment — Submitted filename: Reviewers response.docx [file pbio.3001855.s022.docx]

Rev. 1:
The authors have addressed all the reviewer's concerns and i feel the paper is ready for publication, after just a couple of minor text changes (see below). I particularly like the fact the authors have included "negative" data, which is refreshing to see. I think the paper is nicely written and the results are clearly described without being over-hyped. The paper is addressing an important question and while there are still questions to answer I feel the authors have done enough for publication in PlosBiology.

Minor comments
1) In the intro, the authors could also state that plus end growth from the soma into the axon can also be a mechanism that contributes to plus-end-out polarity in axons and cite Mukherjee et al., 2020.

We added this mechanism and reference

2) When the authors say: "For example, Golgi outposts and early endosomes have been shown to nucleate microtubules in Drosophila dendrites and were suggested to contribute to the minus-end out microtubule organization in these neurites (Mukherjee et al., 2020; Ori-McKenney et al., 2012; Weiner et al., 2020; Ye et al., 2007)". The Mukherjee 2020 paper did not actually show nucleation from Golgi outposts, but rather than most Golgi outposts lacked g-tubulin. Instead, the authors might want to cite papers from the Wildonger lab that suggest mts can be nucleated from Golgi outposts, albeit in a g-tub independent manner.

We corrected this and slightly modified the text to better represent the literature.

Rev. 2:
Some of the points previously raised by reviewers have been addressed satisfactorily, however, there are still some concerns, and some points were not adequately addressed (see below). Overall, the manuscript describes some interesting phenotypes and relationships. However, the new interaction experiments performed did not add mechanistic insight into how the players work together, and the final model : Altogether these results argue against kinesin-1 transport over CAMSAP stabilized microtubules and instead suggests that the MTOC vesicles and the CAMSAP puncta are somehow connected" is somewhat unsatisfying- although I am glad that the authors clearly state here that they do not really know what the connection. At minimum, it is essential to better introduce the new protein, Noca-2, at the center of this story (see below). It might be good idea to consult with an evolutionary biologist and map out the relationships in a meaningful way that will provide a good foundation for future work on Noca/ninein proteins.

Major point
The relationship between Noca-1, Noca-2 and ninein is still not clearly described. From the new phylogeny and region diagram in Supplemental Figure 2B and C it looks like Noca-1 and Noca-2 are similar to different regions of the much longer human ninein? The implication is that Noca-1 and Noca-1 have no similarity to one another. It also means that the phylogeny presented in Figure 2B is not done correctly as it does not show evolutionary relationships between similar regions of a protein. My guess based on the information that is presented is that the large ninein protein present in humans is broken into two separate genes in worms. From the information presented it is impossible to tell whether the ancestral form has both regions in one protein, or whether the two pieces were put together in the vertebrate lineage. As a key finding from this paper is the claim that there are two Noca genes in C. elegans, and one is newly described, the relationships between Noca-1, Noca-2 and ninein are essential to get right, and I am not convinced that the current approach is giving an accurate, clear, or informative overview of how the new gene fits into the family.

As suggested, we consulted with an evolutionary biologist to better describe the relation between NOCA-1, NOCA-2 and NINEIN. He found that indeed NOCA-2 is clearly the NINEIN homologue. Moreover he found that NOCA-1 is the likely CEP85 homologue. This information is now added in the manuscript in Supplementary Figure 2. Accordingly we removed NOCA-1 from the NINEIN phylogenetic tree.

Minor points
Some of the references are used inaccurately, and may mislead other readers. It will be important to double check that all references actually contain the information they are linked to. Here are some examples:s

I think one of the references in this sentence in the introduction is incorrect: "In mammals, Augmin mediated microtubule nucleation and various MAPs were shown to contribute to the mixed microtubule organization (Cunha-Ferreira et al., 2018; Kapitein and Hoogenraad, 2015; Maniar et al., 2011; Sánchez-Huertas et al., 2016; Yau et al., 2014)." I believe the Maniar paper does not include any mammalian data.

I am also not sure that all of the papers referenced in the following sentence demonstrate microtubule nucleation on organelles in dendrites: "For example, Golgi outposts and early endosomes have been shown to nucleate microtubules in Drosophila dendrites and were suggested to contribute to the minus-end out microtubule organization in these neurites (Mukherjee et al., 2020; Ori-McKenney et al., 2012; Weiner et al., 2020; Ye et al., 2007)." For example, I don't think there is any information on nucleation in the Ye paper. And while the Mukherjee paper has some information about Golgi and potential nucleation in the cell body I do not recall any link between dendritic Golgi and nucleation.

While editing the manuscript, the references were not carefully checked. We are sorry for this negligence and thank the reviewer for pointing this out.
